# Modeling Somatic Evolution in Tumorigenesis

**DOI:** 10.1371/journal.pcbi.0020108

**Published:** 2006-08-18

**Authors:** Sabrina L Spencer, Ryan A Gerety, Kenneth J Pienta, Stephanie Forrest

**Affiliations:** 1 Computational and Systems Biology, Massachusetts Institute of Technology, Cambridge, Massachusetts, United States of America; 2 Department of Computer Science, University of New Mexico, Albuquerque, New Mexico, United States of America; 3 Department of Medical Oncology, University of Michigan School of Medicine, Ann Arbor, Michigan, United States of America; 4 Santa Fe Institute, Santa Fe, New Mexico, United States of America; Lawrence Berkeley National Laboratory, United States of America

## Abstract

Tumorigenesis in humans is thought to be a multistep process where certain mutations confer a selective advantage, allowing lineages derived from the mutated cell to outcompete other cells. Although molecular cell biology has substantially advanced cancer research, our understanding of the evolutionary dynamics that govern tumorigenesis is limited. This paper analyzes the computational implications of cancer progression presented by Hanahan and Weinberg in *The Hallmarks of Cancer*. We model the complexities of tumor progression as a small set of underlying rules that govern the transformation of normal cells to tumor cells. The rules are implemented in a stochastic multistep model. The model predicts that (i) early-onset cancers proceed through a different sequence of mutation acquisition than late-onset cancers; (ii) tumor heterogeneity varies with acquisition of *genetic instability,* mutation pathway, and selective pressures during tumorigenesis; (iii) there exists an optimal initial telomere length which lowers cancer incidence and raises time of cancer onset; and (iv) the ability to initiate angiogenesis is an important stage-setting mutation, which is often exploited by other cells. The model offers insight into how the sequence of acquired mutations affects the timing and cellular makeup of the resulting tumor and how the cellular-level population dynamics drive neoplastic evolution.

## Synopsis

Cancer can be viewed as an ecological system in which cells with different mutations compete for survival. In this work, the authors present a three-dimensional stochastic model of these complex interactions. Each cell is represented as an autonomous agent that follows simple rules governing its behavior, where behaviors change as cells gain cancerous mutations. The paper explores the timing of cancer onset, the order in which mutations are acquired, the diversity of tumors, and the competition and cooperation between cells in the tumor microenvironment. One key finding is that early-onset and late-onset tumors take different mutational paths to cancer. The paper provides insight into the early dynamics of tumorigenesis currently inaccessible to experimental investigation.

## Introduction

Cancer progression is a form of somatic evolution in which certain mutations give a cell a selective proliferation advantage [1]. Evidence strongly supports mutation as one of the dominant factors in setting rate-limiting steps in tumor progression, resulting in variation in the timing of progression between tumors [2]. Tumorigenesis is thought to require four to six stochastic rate-limiting mutation events to occur in the lineage of one cell [3–5]. Hanahan and Weinberg suggest that six cellular alterations, or hallmarks, collectively drive a population of normal cells to become a cancer [6]. The six hallmarks are (i) *self-sufficiency in growth signals (SG),* (ii) *insensitivity to antigrowth signals (IA),* (iii) *evasion of apoptosis (EA),* (iv) *limitless replicative potential (LR),* (v) *sustained angiogenesis (SA),* and (vi) *tissue invasion and metastasis*. *Genetic instability (GI)* is defined as an “enabling characteristic” that facilitates the acquisition of other mutations due to defects in DNA repair. These hallmarks form a candidate set of rules that underlie the transformation of a normal tissue to a cancerous one. The quantitative ramifications of these rules are explored in this paper, and lead to a number of interesting phenomena and hypotheses.

We model a simplified view of cancer progression using a stochastic model of tumorigenesis based on the hallmarks. The complexity of cancer cannot be understood by considering individual mutations independent of their interactions. Rather, the effect of a mutation often depends on other mutations within the same cell, on other mutant cells within the tumor, and on the tumor microenvironment. Our model allows us to study the evolutionary dynamics of early mutations, which generally go undetected in clinical settings, and thereby examine the initial forces that drive neoplastic evolution.

## Materials and Methods

This paper extends earlier work using the hallmarks of cancer to study cancer progression [7,8]. Here, we model the hallmarks phenotypically (one mutation, one hallmark) in a three-dimensional, agent-based model that resembles a stochastic cellular automaton [8]. The computational grid contains a maximum of 10^6^ cells (100 × 100 × 100) initialized with a single normal cell and a single blood source. As normal proliferation occurs, cells far from the blood supply signal for angiogenesis. The blood supply then extends in the direction of the requesting cell, creating a branching pattern of vascularization, which may pass through a cube with or without a resident cell. The nutrient available to each cell is the sum of the contributions from all capillaries. However, distant capillaries contribute little, as nutrient levels fall as a power law function of the distance from their source. This signaling and vascularization process continues until the cells fill the space established by two intersecting three-dimensional parabolas that represent angiogenic and growth factor constraints. Normal cells are able to signal for vascularization until they reach the angiogenic boundary, beyond which they are not able to signal for the resources needed to divide. Similarly, normal cells are able to divide only within the region containing growth factor. This intersection restricts normal tissue to 8.6 × 10^4^ cells.

The event cycle for each cell defines the progression of the model. This cycle has a period defined as a random number drawn from a uniform distribution between five and ten time steps. At the beginning of this cycle, a normal cell will initiate cell division if there is unoccupied adjacent space. The parent cell “genome” is copied with a small probability of mutation (1/*m*), the telomere length (*t*) of both cells is decreased by a single unit, and the new daughter cell fills the empty neighboring space. If there is no adjacent space for a daughter cell, a normal cell will remain quiescent until the next cycle. During this cycle there is a small probability of random death (*a*) and a probability of death associated with acquired mutations (*n/e*, discussed below). This process continues until either 50,000 time steps have passed or the tissue has developed cancer. We define the onset of “cancer” to occur when the tissue grows beyond the normal tissue boundary to occupy 90% of the grid, or 9 × 10^5^ cells, at which point the run terminates. As described in the first two sections under Results, we performed 1,000 runs of the model with the default parameter settings described below. Of these, 986 end in cancer before 50,000 time steps.

Normal cells require mitogenic growth signals to switch from a quiescent to a proliferative state. Cancer cells generate many of their own growth signals and are less dependent on exogenous stimulation. For example, activation of the Ras oncogene allows a cell to send ongoing mitogenic signals even without stimulation of the upstream regulators [6]. In the model, normal cells divide only if they are within the predefined spatial boundary that represents the growth factor concentration threshold. The acquired mutation *SG* allows cells to proliferate regardless of the concentration of growth factor. Therefore, this mutation is necessary for cells to proliferate beyond the normal tissue boundary.

In normal tissue, antigrowth signals maintain cells in a nondividing state. These antigrowth signals are often proliferation inhibitors that force cells into a quiescent or post-mitotic state by converging on the retinoblastoma protein, pRb, which controls progression from *G*_1_ into S phase. Cancer cells can also downregulate the expression of cell adhesion molecules that send antigrowth signals [6]. Contact inhibition is a type of antigrowth signal that prevents cell division if a cell is surrounded by many other cells, thus preventing overcrowding. In the model, each cell has up to 26 neighbors (neighbors need only make contact at a corner). Normal cells do not divide if all 26 positions are occupied by other cells. The mutation *IA* allows cells to divide even when there is no space for the daughter cell. The daughter cell competes for survival with a randomly chosen neighbor with a 1/*g* chance of success. If successful, the daughter cell replaces the randomly chosen cell. The model could include structural changes to the tissue, such as crowding or expansion pressures. However, these issues and their associated assumptions about tissue architecture go beyond the intended scope of this model.

Cell populations are able to expand in number by inappropriate cell division as well as by lack of appropriate cell death (apoptosis). A cell monitors its own state and initiates apoptosis in response to signals such as DNA damage, oncogene overexpression, survival factor insufficiency, or hypoxia [6,9]. Mutations to genes involved in apoptosis can change the balance between pro-death and pro-survival signals. For example, upregulation of the anti-apoptotic protein Bcl-2 can shift the signaling balance toward survival [6]. In the model, cells are checked for the presence of mutations each cell cycle, and if a mutation is detected, the cell is eliminated. The probability of detecting genetic damage in the cell is *n/e*, where *n* = 0,...,6 is the number of mutations carried by the cell, and *e* is the probability of death. The mutation *EA* allows a cell to evade mutation detection and the subsequent death that would usually occur.

In cell culture, telomere shortening limits normal human cells to 25–70 doublings [6,10]. However, 80% of human cancers show expression and activity of telomerase, an enzyme that lengthens telomeres and confers limitless replicative potential [11]. In the model, the initial cell is created with telomeres of length *t,* where *t* is 50 unless otherwise specified. At each cell division, the telomere length is decreased by one. When telomere length reaches zero in the model, the cell dies. The mutation *LR* allows the cell to divide indefinitely. The implications of this simplification are discussed in detail in the Results section.

Cells cannot survive at distances of more than 100 *μm* from a blood supply, which limits the size of human tumors to about 10^6^ cells without angiogenesis [12]. For an incipient tumor to grow, cells must reinstate the angiogenic ability that was temporarily present during organogenesis. For example, when the concentration of nutrients and oxygen is low, cancer cells increase production of the pro-angiogenic signal, vascular endothelial growth factor [6]. In the model, lack of oncogenic angiogenesis limits tumorigenesis until the mutation *SA* confers the ability to signal for growth of new blood vessels. Once cells acquire this mutation, they are able to signal for angiogenesis outside the previously established vascular system. This allows cells to acquire the nutrients necessary to expand beyond the normal tissue boundaries.

Cells maintain genomic and karyotypic integrity through a complex set of DNA monitoring proteins, DNA repair enzymes, and mitotic checkpoint proteins. Thus, the mutation rate in human cells is relatively low, estimated to be in the range of 10^−7^ to 10^−6^ mutations/gene/cell division [13]. We define the mutation rate to be 1/*m* and choose *m* as the midpoint between 10^7^ and 10^6^, such that *m* = 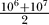 = 5.5 × 10^6^. Loss of DNA repair signaling can increase the mutation rate by a factor ranging from 10^1^ to 10^4^ [14]. In the model, cells that gain the mutation *GI* have their base mutation rate scaled up by *i*. We chose a midrange value, defining *i* as 10^3^.

We assume that cells experience some amount of random death due to causes not previously specified, such as injury or stress. In the model, every cell has a 1/*a* likelihood of dying at some point in the interval described by the uniform distribution discussed above. The three parameters *g*, *e*, and *a* were chosen via an informal sensitivity analysis such that the parameter plays an important role but does not dominate tumorigenesis. We selected *g* to be 5, *e* to be 20, and *a* to be 1,000. Finally, we do not model tissue invasion and metastasis, as the model represents a single tissue.

When modeling a complex biological process, it is always necessary to make simplifying assumptions. The fundamental concern of this paper is to study cancer at a cell population level, modeling tumors as evolving ecosystems, and not to incorporate every known piece of molecular cell biology relating to cancer. The source code that implements the model and the associated graphical user interface are available online under the terms of the General Public License at http://cs.unm.edu/~forrest/software/cancersim.

## Results

### Pathways to Cancer Vary with the Timing of Cancer Onset

The standard perspective on tumorigenesis suggests that cells derived from the same lineage acquire multiple mutations in discrete steps [6]. We define the sequence of mutation acquisition as a “pathway” to cancer. With *n* = 6 different mutations possible and a variable number of mutations within each cell, there are 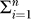 *_n_P_i_* = 1,956 distinct sequences of mutation history. We are interested in the pathways cells take to become a tumor, and how particular pathways affect the dynamics of tumorigenesis. It is currently believed that it is not simply the accumulation of mutations, but also the order in which they are acquired, that determines the timing and extent of tumorigenesis [6,15]. The model allows us to study the sequence in which mutations are accumulated. We find that the pathways leading to early-onset cancer (arising early in the life of the simulated tissue) are different from the pathways leading to late-onset cancer.

The importance of genetic instability in cancer progression is a controversial issue in cancer biology. Loeb and others argue that early acquisition of a mutator phenotype is necessary for tumorigenesis [16,17], while Tomlinson, Bodmer, and others believe that increased cell division provides sufficient opportunities for mutation accumulation [18–20]. Although this question must ultimately be answered empirically, our model informs this debate in a new way by proposing a compromise between these two opposing theoretical viewpoints.

**Figure 1:**
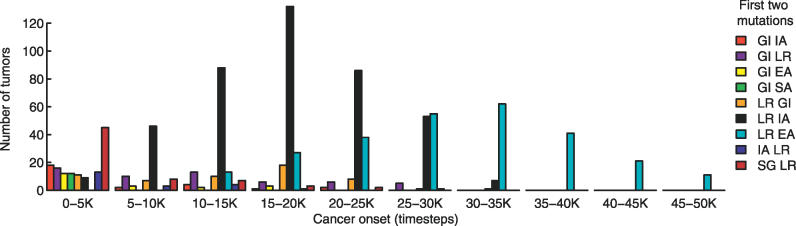
The Timing of Cancer Onset Is Correlated with the Most Common First Two Mutations of a Tumor The most common first two mutations of a tumor define the dominant tumor type. *GI* is the most common first mutation in the 0–5 K timeblock, as seen by summing the populations with *GI* as the first mutation. The prevalence of *GI IA, GI EA, GI SA, IA LR,* and *SG LR,* decreases after 5,000 time steps, while *GI LR* and *LR GI* remain roughly constant for several time blocks. The sequences *LR IA* and *LR EA* are distributed around 15–20 K and 30–35 K time steps, respectively. Dominant tumor types not shown occur in less than 2% of the 986 runs ending in cancer.

**Figure 2:**
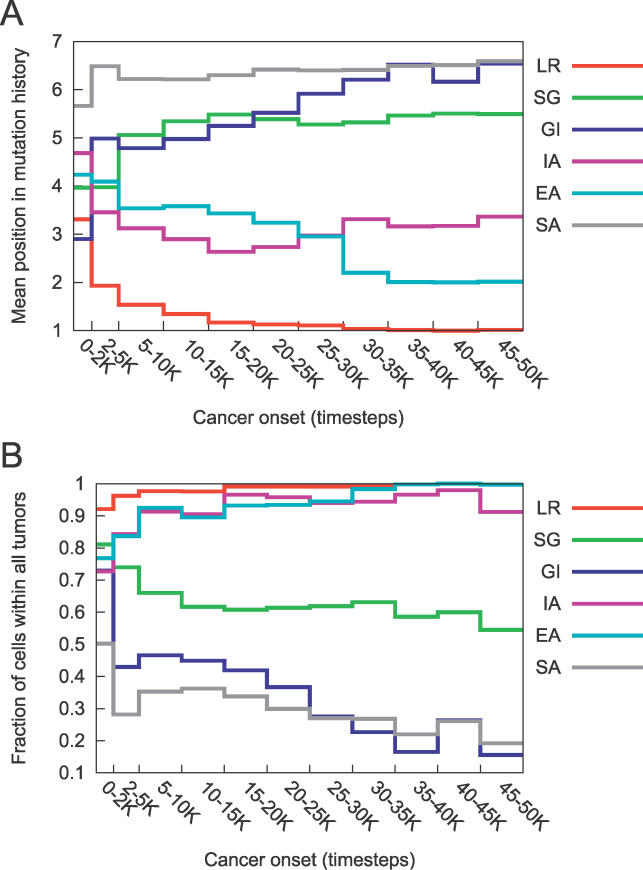
Pathways to Cancer Vary with the Timing of Cancer Onset (A) Average position of the mutations in the pathways present in the 986 runs that terminated in cancer, for tumors acquired in the timeblocks specified on the *x*-axis. A position of one indicates that it was the first mutation of a pathway. A position of seven indicates that a given mutation did not appear in the pathway. For cancers arising before 2,000 time steps, *GI* was frequently the first mutation, as it has the lowest mean position. As time progresses and telomere length shortens, *LR* becomes the first mutation in all pathways. Standard deviation and sample size data are included in Table S2. (B) Fraction of all cells in all tumors carrying a given mutation, in which cancer onset occurred in the timeblocks specified on the *x*-axis. Cells with mutations in *LR, IA,* and *EA* make up the majority of the tumors. The frequency of *GI* drops substantially as time of cancer onset increases, whereas the frequency of *SA* remains low for all timeblocks.

We find that *GI* is often the causative mutation in earlier onset tumors, while *LR* is often the causative mutation in later onset tumors (Figure 1, Figure 2A). Figure 2A shows that very early tumors (before 2,000 time steps) frequently result from acquiring *GI* as the first mutation. This can also be seen by defining the dominant tumor type to be the most common first two mutations in a given tumor. Figure 1 displays the frequency of each dominant tumor type as a function of the cancer onset time. As indicated in the figure, *GI* is the most common first mutation in the 0–5 K timeblock, as seen by summing the populations with *GI* as the first mutation. This is consistent with the fact that many early-onset cancers arise from inherited mutations in genes affecting the stability of the genome. These include xeroderma pigmentosum, ataxia telangiectasia, Nijmegen breakage syndrome, and Bloom syndrome [20]. Conversely, tumors acquiring *GI* late in their pathway gain little selective advantage, as the cells have already acquired most necessary mutations. Thus, in tumors where *GI* occurs late in the pathway (Figure 2A), the frequency of this mutation is relatively low (Figure 2B).

As indicated by Figure 1, a tumor is unlikely to form early (prior to 5,000 time steps) without either the increase in mutation rate associated with *GI* or a rapid proliferation of cells. For example, in the rare case that a cell on the tissue boundary acquires SG, the subsequent rapid clonal expansion provides ample opportunity for further mutations. This is illustrated by the prevalence of *SG* as a first mutation in early tumors.

Later onset tumors in the model, however, are initiated by the acquisition of *LR* followed by either *IA* or *EA* (Figure 1, see *LR IA* and *LR EA*, and Figure 2). For later onset tumors, the many rounds of cell division offer sufficient opportunity for mutation accumulation. However, by limiting the lifespan of the cell, telomere shortening prevents acquisition of a sufficient number of mutations. Without *LR,* mutations accumulate for only a limited number of cell divisions. Acquisition of *LR* immortalizes cells, allowing them to maintain a mutational line over the lifetime of the tissue. Clinical evidence supports our finding of *LR* as a relatively early event in tumor development, demonstrated by the presence of telomerase activity in many precancerous lesions (reviewed in [21]).

Additionally, we find that *EA* is an important mutation to gain early in the pathway, regardless of when the tumor arises, as it prevents detection of acquired mutations (see Figure 2A). Indeed, it has been noted in the literature that evasion of apoptosis is not inherently mutagenic at the cellular level. Rather, the mutation allows for the perpetuation of mutant cells that would otherwise be removed, resulting in higher numbers of mutations at the population level [22].

The model underscores the importance of accumulating mutations in an individual cell. Due to the low probability of acquiring multiple mutations in a single cell, either the mutation rate must increase through *GI,* or *LR* must allow cells to live long enough to acquire multiple mutations. Thus, *GI* and *LR* were either the first or second mutation in the most common pathways of all but three of the 986 observed tumors (data shown in Table S1). Thus, the model suggests the following compromise in the mutator phenotype debate: genetic instability plays a key role in early-onset tumors, while for later tumors, limitless replicative potential provides sufficient opportunity to acquire an equal number of mutations by allowing an increased number of cell divisions.

### Heterogeneity Varies with the Predominance of *GI,* Mutation Pathway, and Selective Pressures during Tumorigenesis

Tumor heterogeneity has important consequences for the progression and treatment of cancer [23]. For example, a recent paper finds that clonal diversity is a good predictor for progression to esophageal adenocarcinoma [24]. Our model makes three predictions: (i) tumor heterogeneity is strongly correlated with the predominance of *GI* within the tumor; (ii) tumors with distinct causative mutation pathways exhibit distinct levels of heterogeneity; (iii) during tumor progression there is often a marked increase in heterogeneity followed by a less dramatic decline.

We study heterogeneity using a diversity measure adapted from ecology and evolutionary biology [25]. The metric defines heterogeneity as the average pathway distance between all pairs of cells of a given tumor. Pathway distance is measured as the total number of steps backward through the lineage tree required to reach a common mutation history added over both lineages. For instance, a cell with pathway *LR EA GI* and a cell with *LR EA SG* would have a pathway distance of two, a cell with pathway *LR EA GI SG* and a cell with *LR EA IA SG* would have a pathway distance of four, and a cell with *LR EA* and a cell with *LR EA GI SG* would have a pathway distance of two. Tumor heterogeneity is defined as the average pathway distance between all pairs of cells:

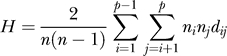
where *n* is the total number of cells in a tumor, *p* is the total number of distinct pathways, *n_i_* is the number of cells with mutation pathway *i, n_j_* is the number of cells with mutation pathway *j,* and *d_ij_* is the pathway distance between the two mutation pathways. Dividing by *n*(*n* − 1)/2 provides the average of this summation. By choosing the pathway as the level of analysis, rather than the set of mutations disregarding order, we capture the process of mutation acquisition.

Our results suggest that tumors initiated by *GI* early in their pathways are more heterogeneous than other tumors. In other words, the position of *GI* in the pathway is negatively correlated with tumor heterogeneity (*p* < 0.001, *R* ^2^ = .46, ordinary least-squares regression). These results are consistent with evidence that points to genetic instability as a source of heterogeneity [24,26]. Additionally, the time of cancer onset remains a significant determinant of heterogeneity (*p* < .05) even when the predominance of *GI* is accounted for.

**Figure 3:**
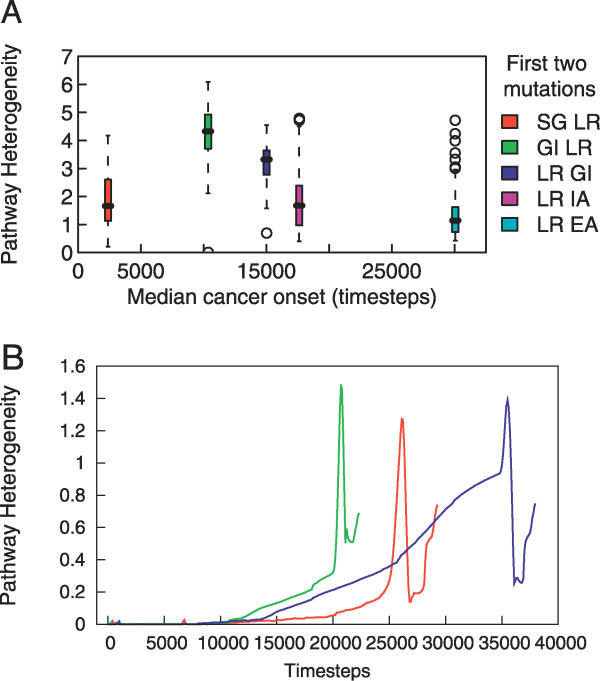
Tumor Heterogeneity (A) The five most common tumor categories, defined by the most common first two mutations, vary in their pathway heterogeneity. (B) Example dynamics of heterogeneity during the development of three tumors. Each line corresponds to the development of one tumor; the red curve corresponds to the tumor discussed in the Sample Simulation Run section. As the tumor begins to form, there is typically a slow increase in the degree of tissue heterogeneity followed by a sudden increase, an equally sudden decrease, and then often another increase as the tissue reaches 9 × 10^5^ cells.

Except for the pair *LR IA* and *SG LR*, the five most common tumor categories, as defined by their most common initial two mutations, have statistically distinct levels of heterogeneity in pair-wise comparisons (*p* < 0.001, *t*-test). The boxplot in Figure 3A shows the distributions of heterogeneity among various tumor groups. Interestingly, *GI LR* and *LR GI* tumors have the highest levels of heterogeneity.

A preliminary analysis of the relationship between tumorigenesis and heterogeneity reveals an interesting phenomenon. During tumorigenesis, there is a marked increase in intra-tumor heterogeneity. In many tumors, the level of heterogeneity peaks, declines, and then rises again as the tissue becomes a tumor. Figure 3B illustrates this pattern in three tumors. This suggests that rapid proliferation allows for increased diversity upon which selection can act to choose the fittest cells, leading to a subsequent decrease in heterogeneity. The dynamic balance between the selective advantage of diversity and the selective pressure to homogenize may underlie a complex relationship between heterogeneity and tumorigenesis, which may have important clinical implications.

### Initial Telomere Length Affects Cancer Onset Time and Incidence

Limited replication protects against indefinite proliferation of mutant cell lines. A majority of human cancers show expression and activity of telomerase [11], an enzyme that lengthens telomeres. Expression of telomerase is not found in most normal somatic tissues and confers unlimited replicative potential. Lengthened telomeres are thought to facilitate tumorigenesis by allowing cell lines to accumulate the mutations necessary to develop a tumor [6]. However, short telomeres can also promote tumorigenesis by creating chromosomal instability [21]. For instance, there is evidence that short telomeres are associated with an increase in malignancy in mice due to the resulting genomic instability [27,28].

Karyotypic instability resulting from telomere shortening represents a deviation from normal cell behavior. Typically, normal cells with shortened telomeres stop dividing and enter senescence. However, cells with dysfunctional pRb and p53 tumor suppressor proteins are able to avoid senescence. These cells continue to proliferate until the fusion of the now naked chromosomal ends results in crisis, a state marked by widespread cell death due to karyotypic instability. A rare mutational event may activate telomerase, stabilizing the chromosomal ends, and giving the cell's lineage the selective advantage of immortality [6].

Just as long and short telomeres can both contribute to tumorigenesis, our model reveals a similar tradeoff between long and short telomere lengths. However, we note that this finding may depend on an important simplification. To avoid complicating the model's simple representation of cellular aging and to maintain the explicit focus on intercellular rather than intracellular dynamics, senescence, crisis, and karyotypic instability were not modeled. These processes are not well-understood and would significantly increase the number of built-in assumptions. In many ways, telomere shortening deserves a model of its own, since the sequence of events, the contexts in which they occur, and the associated probabilities are complex. Instead, we make a conservative simplification, eliminating cells once their telomere length reaches zero. One might imagine that this approach would neutralize the deleterious effects of shortened telomeres, as these karyotypically unstable cells are prevented from developing. However, even the conservative model produces the same tradeoff between long and short telomere lengths as found in cells.

**Figure 4:**
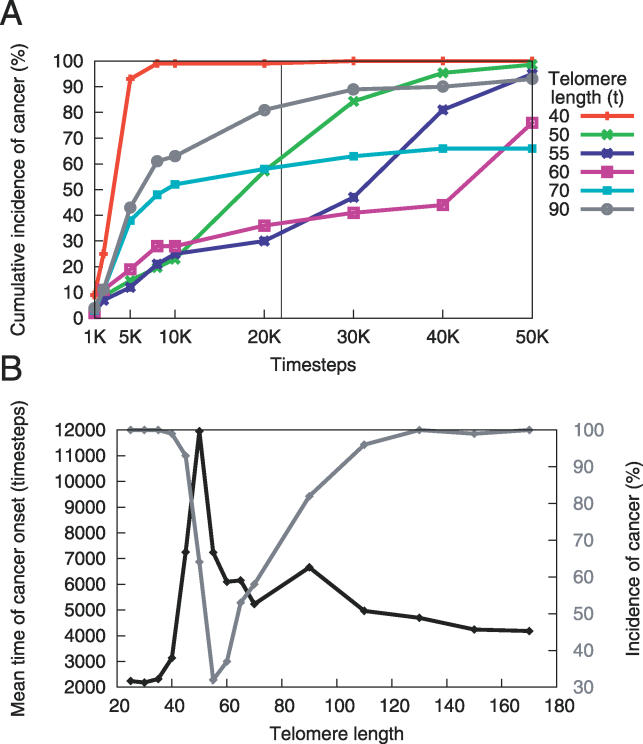
Initial Telomere Length Affects Incidence and Onset of Cancer (A) Initial telomere length affects the pattern of incidence across time. Each point in time on the *x*-axis represents the cumulative incidence of cancers that arose before that time. The initial telomere length governs the tradeoff between the incidence of early and late cancer onset. Short (40 units) and long (90 units) telomeres produce an earlier, higher incidence of cancer than do telomeres of intermediate length. (B) Mean onset time and incidence for cancers acquired before 21,900 time steps as a function of initial telomere length. This subfigure represents a snapshot at time *t* = 21,900, indicated by the vertical black line in (A). Note that the gray curve corresponds to the secondary *y*-axis.

In a separate set of simulations, we vary *t,* the initial telomere length. We find that both long (90–170 units) and short (25–40 units) telomere lengths lead to higher incidence and earlier time of cancer onset than do intermediate lengths (Figure 4). In addition, there exists a tradeoff between low early incidence and higher late incidence of cancer. The initial tumorigenic effects of long telomere length are due to the large replicative potential of the cells. As the cells continue to divide, their telomeres reach an intermediate length where there is lower cancer incidence. After more cell divisions, the cells' telomeres become very short, causing cell death. This increases cancer incidence in the model because additional mutations can occur during the cell divisions that replace the exhausted cells.

The initial telomere length that minimizes cancer incidence at any particular point is a function of the tissue's age. Figure 4B reveals that, with a lifespan of 21,900 time steps, a telomere length of 55 corresponds to the lowest incidence of cancer, and a length of 50 produces the latest mean onset time. Thus, there exists in the model an optimal range of initial telomere lengths that lowers cancer incidence and raises time of cancer onset. This raises the possibility that evolution has played a role in optimizing telomere length.

### Angiogenesis Sets the Stage for Tumor Growth

The composition of the final tumor does not necessarily reflect the complete evolutionary dynamics by which it was produced. *SA* is an example of a stage-setting mutation that is essential for tumorigenesis but which can be exploited by other cells. Once the blood supply is established, angiogenic cells without other selective advantages can experience limited clonal expansion or be driven to extinction. Clonal expansion is limited by competition from other cells proliferating into the newly vascularized region, often preventing additional angiogenesis.

**Figure 5:**
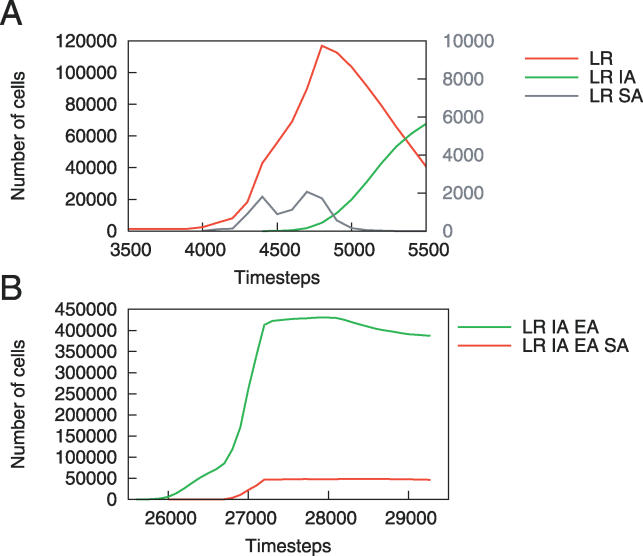
*SA* Creates a Niche for Other Cell Populations (A) A population of *LR SA* cells (secondary *y-*axis) allows the population of *LR* cells to proliferate. Eventually, the *LR IA* cells replace the *LR SA* cells, temporarily preventing the development of new vasculature. (B) In this case, the cells creating the blood supply have additional mutations, allowing the *LR IA EA SA* population to plateau rather than decline, as occurred in the case of the *LR SA* cells in (A).

In some cases, the clonal expansion of cells with *SA* is not only impeded, but the population also completely recedes (Figure 5A). Here, the initial proliferation of a population of *LR SA* cells facilitates the expansion of *LR* cells and then declines. A second rise in this population of *LR SA* cells then supports the proliferation of *LR IA* cells, which “free ride” on the *LR SA* population, quickly driving the population of angiogenic cells to extinction. The precancerous cells' lack of coordination results in the decline of this *LR SA* population and considerably slows tumorigenesis.

Figure 5B illustrates the formation of a new evolutionary niche by a population with *LR IA EA SA,* allowing neighboring *LR IA EA* cells to proliferate, too. The population of *LR IA EA* cells initially expands as blood supply becomes available. However, as angiogenesis is completed within the region, the selective advantage of the *SA* mutation disappears and the angiogenic cells are constrained by other cells. In this situation, the *LR IA EA SA* cells creating the blood supply have two additional mutations, which allow them to compete more successfully than the *LR SA* cells did in Figure 5A. Thus, the angiogenic population is able to maintain itself rather than be driven to extinction. Due to the fact that a few cells with an *SA* mutation provide a collective benefit to all cells nearby, and that once a blood supply is established the angiogenic cells lose their selective advantage, this mutation rarely occurs in the pathways composing the final tumor (Figure 2). Beyond providing nutrients as a direct collective benefit, increased angiogenesis also allows for increased clonal expansion, providing additional opportunities for new mutations to be acquired.

### Sample Simulation Run

Due to the stochastic nature of the model, each run displays different cell population dynamics. Here, we describe the dynamics of a representative run for the parameters described in the Materials and Methods section, and a random seed of three.

**Figure 6:**
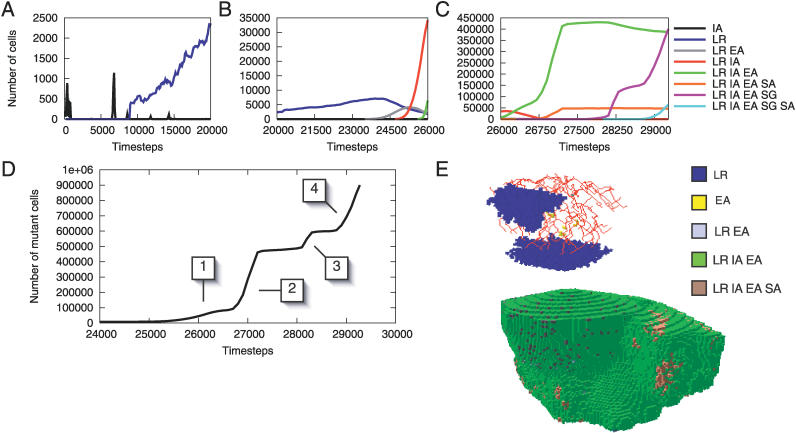
Cell Dynamics of the Sample Run Top row: growth dynamics of mutant cells in three graphs corresponding to three different time periods. (A) Populations with *IA* rise and fall, and cells with *LR* emerge. (B) Around time step 24,000, a cell with *LR EA* undergoes clonal expansion, resulting in a decline of the parent *LR* population. Near time step 25,000, cells with *LR IA* begin to outcompete the *LR EA* population. (C) At time step 26,000, cells with *LR IA EA* expand while the *LR IA* population declines. The emergence of a clonal population with *LR IA EA SA* provides the angiogenesis for the *LR IA EA* population to expand rapidly. At time step 28,000, *LR IA EA SG* cells begin to double the size of the tumor, aided by the *LR IA EA SG SA* cells. (D) The aggregate cell proliferation pattern in mutant cells. The boxed numbers indicate clonal expansions that result from overcoming proliferation bottlenecks. The first expansion occurs with the acquisition of IA. The second expansion is the result of an *SA* mutation. The third occurs with the acquisition of SG, and the final expansion occurs with the acquisition of SA. (E) The tissue at time step 20,780 (top) and 26,982 (bottom). Normal cells have been removed from the image to reveal two clonal populations with *LR* at time step 20,780. At time step 26,982, the tumor has grown beyond the normal tissue extent through the acquisition of SA.

Several populations of cells with *IA* independently arise and die out in the early stages of the run (Figure 6A). At time step 8,888, a cell acquires *LR* and begins to spread (Figure 6A). This is the first cell lineage to arise that remains in the tissue until the end of the run, and thus can be considered a “causative” tumorigenic mutation. At time step 20,176, another cell independently develops *LR* and begins to spread. A snapshot of the tumor at time step 20,780 show these two separate colonies (Figure 6E, top). Shortly after time step 23,420, four populations of cells with *LR EA* emerge within the first population of *LR* cells. The slight fitness advantage conferred by the mutation allows slow clonal expansion and a decline in the parent *LR* population (Figure 6B). During this expansion, a cell with *LR* gains *IA* and begins to proliferate. Although the *IA* mutation alone was previously unsuccessful, the combination of *LR* and *IA* allows these cells to proliferate rapidly. Quickly, the population with *LR IA* begins to dominate other cells. This expansion allows several *LR IA* cells to independently gain EA, becoming *LR IA EA* cells. Ultimately, this population takes over the normal tissue and replaces the previous mutant populations (Figure 6C). Many cells acquire additional mutations, but they remain limited in population size.

The population of cells expands beyond the normal tissue boundary when one of the cells gains *SA* and signals for vascularization outside the tissue's natural extent, allowing the population to proliferate in a previously unoccupied region. The *LR IA EA* cells exploit the angiogenesis, successfully competing for the new habitable region (Figure 6E, bottom). Clonal expansion is limited until a cell near the bounded region acquires SG. When this occurs, the population of *LR IA EA SG* cells expands (Figure 6C), and once this population gains SA, the tumor grows rapidly in size until it reaches 9 × 10^5^ cells at time step 29,272, and the run ends.

During tumorigenesis, the tissue experiences a number of rate-limiting steps (Figure 6D). Each passage through a bottleneck corresponds to acquisition of a mutation in the sample run. The pathway heterogeneity within the tissue gradually increases until time step 25,000 (red curve, Figure 3B), at which point there is a dramatic increase in heterogeneity corresponding to the beginning of a gradual increase in the number of mutant cells (Figure 6D, [1]). The tumor initially grows within the normal tissue's natural extent after acquisition of *IA* [1]. The acquisition of *SA* allows for rapid expansion beyond this region [2]. This growth is limited until a population with *SG* clonally expands [3], which is in turn limited until further *SA* mutations occur [4].

In general, *GI* and *LR* are frequently the causative mutations in very early-onset and later-onset cancers, respectively, but *SA* and *SG* are key for providing transitions through proliferation bottlenecks. Targeted therapies in cancer treatment are based on identifying molecular bottlenecks and exploiting them for therapeutic intervention. For example, the vascular endothelial growth factor inhibitor bevacizumab is an antibody that binds to the growth factor and prevents it from binding to its receptor. Bevacizumab is approved for use in colon cancer and is a prototypical example of a targeted therapy based on a tumorigenesis bottleneck [29].

## Discussion

The key findings from this paper are the following four. (i) Early-onset cancers proceed through a different sequence of mutation acquisition than late-onset cancers. Specifically, *GI* is the most common first mutation in early-onset cancers whereas *LR* is the most common first mutation in later-onset cancers. (ii) Heterogeneity varies with early acquisition of *GI,* mutation pathway, and selective pressures during tumorigenesis. (iii) There exists a range of optimal initial telomere lengths that lowers cancer incidence and raises the time of cancer onset. (iv) The ability to initiate angiogenesis is an important stage-setting mutation, which is often exploited by other cells and is therefore infrequently present in final tumors.

This model presents a first step toward predicting the fate of early precancerous mutations computationally. Early events responsible for neoplastic progression are difficult to investigate experimentally for the very reason that they have not yet been detected. The main limitation of the model is the difficulty of experimental validation. A thorough testing of the model would require periodically examining single cells for the presence of mutations in the hallmark categories, beginning before an animal has developed a clinically detectable cancer. Initiating testing for mutations once the animal has a palpable tumor ignores early mutation dynamics that, according to the model, are important for determining the timing and cellular makeup of the tumor that develops. In addition, the current technology of sequencing populations of tumor cells for mutations at various timepoints does not provide explicit support of the multistep model of tumorigenesis, which requires multiple mutations to be found in a single cell.

The work presented here relies on a number of simplifying assumptions, the most important relating to tissue architecture and molecular intracellular processes. For instance, we assume that all mutations fit into one and only one of the six hallmarks, whereas p53, for example, is known to be involved in cell cycle inhibition, apoptosis, genetic stability, and inhibition of blood vessel formation [30]. However, we simplified the mapping because disaggregating the hallmarks allowed us to better study their interactions. Modeling simultaneous multiple hallmark acquisition would require knowledge of which combinations are possible and their probabilities of occurring. For example, although p53 is involved in several hallmarks, not every mutation to this protein causes the complete loss of p53 functionality. Additionally, as discussed in the Results section, a more complex model of cellular aging would more accurately reflect known biology. An advantage of our agent-based model is that alternative approaches can be easily tested by downloading and modifying the code that runs the model. Future work could address the above issues, and could implement mutations such as *GI* as a continuum rather than as a binary switch. Future research could also include extending the model to incorporate deleterious mutations to housekeeping genes, invasion and metastasis, and cancer stem cells.

Although simplifications are inevitable in a theoretical model, this work nevertheless reveals important consequences of the well-established concepts proposed by Hanahan and Weinberg. This type of model can uncover unexpected nonlinear interactions, such as those we find among the six hallmarks. The model provides insight into the early dynamics of neoplasia currently inaccessible to experimental investigation and thus serves as a tool for hypothesis generation.

## Supporting Information

Table S1 The Number of Tumors as Categorized by the Most Common First Two Mutations (22 KB PDF)

Table S2 Mean, Standard Deviation, and Sample Size for Data in Figure 2A (15 KB PDF)

### Accession Numbers

The Entrez Gene (http://www.ncbi.nlm.nih.gov/entrez) GeneID accession numbers for the genes and gene products discussed in this paper are: BCL2 (GeneID 596), KRAS (GeneID 3845), RB1 (GeneID 5925), TERT (GeneID 7015), TP53 (GeneID 7157), and VEGF (GeneID 7422).

## Abbreviations

*EA*: *evasion of apoptosis*
*GI*: *genetic instability*
*IA*: *insensitivity to antigrowth signals*
*LR*: *limitless replicative potential*
*SA*: *sustained angiogenesis*
*SG*: *self-sufficiency in growth signals*
