# Supplementary material for: Modeling Somatic Evolution in Tumorigenesis
Source: PLoS Comput Biol. 2006 Aug 18;2(8):e108. doi: 10.1371/journal.pcbi.0020108 (PMC1550273; doi:10.1371/journal.pcbi.0020108)
Supplement: Supplementary file 1 [file pcbi.0020108.st001.pdf]

Table S1: The number of tumors as categorized by the most common first two mutations. Bold indicates tumors that do not have *LR* or *GI* as a first or second mutation.

Most common first two mutations	Number of tumors
LR IA	421
LR EA	268
SG LR	65
GI LR	56
LR GI	56
GI IA	27
IA LR	22
GI EA	20
LR SG	16
GI SA	12
LR SA	11
GI SG	4
SG GI	3
**EA IA**	**1**
**IA EA**	**1**
IA GI	1
SA LR	1
**SG EA**	**1**
