# Supplementary material for: Modeling Somatic Evolution in Tumorigenesis
Source: PLoS Comput Biol. 2006 Aug 18;2(8):e108. doi: 10.1371/journal.pcbi.0020108 (PMC1550273; doi:10.1371/journal.pcbi.0020108)
Supplement: Supplementary file 2 [file pcbi.0020108.st002.pdf]

Table S2: Mean, standard deviation, and sample size for data in Figure 2A

Timeblock	Average position (std)						# of tumors
	LR	SG	GI	IA	EA	SA	
0-2K	3.31 (0.38)	3.97 (0.46)	2.90 (0.49)	4.68 (0.59)	4.23 (0.56)	5.66 (0.67)	86
2-5K	1.93 (0.24)	3.97 (0.46)	4.99 (0.71)	3.46 (0.44)	4.09 (0.49)	6.48 (0.78)	60
5K-10K	1.54 (0.18)	5.06 (0.60)	4.79 (0.71)	3.12 (0.39)	3.54 (0.42)	6.22 (0.77)	84
10K-15K	1.34 (0.17)	5.35 (0.64)	4.97 (0.68)	2.89 (0.36)	3.58 (0.43)	6.21 (0.75)	148
15K-20K	1.17 (0.15)	5.48 (0.68)	5.25 (0.72)	2.64 (0.32)	3.43 (0.41)	6.30 (0.78)	194
20K-25K	1.13 (0.15)	5.39 (0.69)	5.52 (0.76)	2.73 (0.35)	3.24 (0.40)	6.42 (0.82)	147
25K-30K	1.11 (0.14)	5.28 (0.69)	5.92 (0.85)	2.97 (0.41)	2.96 (0.41)	6.40 (0.88)	124
30K-35K	1.04 (0.15)	5.32 (0.76)	6.21 (0.96)	3.31 (0.48)	2.20 (0.32)	6.41 (0.97)	70
35K-4K	1.01 (0.16)	5.47 (0.86)	6.52 (1.06)	3.16 (0.51)	2.01 (0.31)	6.49 (1.06)	41
40K-45K	1.00 (0.15)	5.51 (0.86)	6.16 (1.02)	3.17 (0.49)	2.00 (0.31)	6.51 (1.05)	21
45K-50K	1.02 (0.14)	5.49 (0.80)	6.54 (0.93)	3.36 (0.47)	2.01 (0.28)	6.59 (0.95)	11
